# H3K27me3 at pericentromeric heterochromatin is a defining feature of the early mouse blastocyst

**DOI:** 10.1038/s41598-022-17730-x

**Published:** 2022-08-16

**Authors:** Mélanie Pailles, Mélanie Hirlemann, Vincent Brochard, Martine Chebrout, Jean-François Oudin, Hendrik Marks, Alice Jouneau, Amélie Bonnet-Garnier

**Affiliations:** 1 Université Paris-Saclay, UVSQ, INRAE, BREED, 78350 Jouy-en-Josas, France; 2 Ecole Nationale Vétérinaire d’Alfort, BREED, 94700 Maisons-Alfort, France; 3 Department of Molecular Biology, Faculty of Science, Radboud Institute for Molecular Life Sciences (RIMLS), Radboud University, 6525GA Nijmegen, The Netherlands

**Keywords:** Embryogenesis, Embryonic stem cells, Epigenetics, Pluripotency

## Abstract

Early mouse development is characterized by structural and epigenetic changes while cells progress towards differentiation. At blastocyst stage, the segregation of the three primordial lineages is accompanied by establishment of differential patterns of DNA methylation and post-translational modifications of histones, such as H3K27me3. Here, we analysed the dynamics of H3K27me3 at pericentromeric heterochromatin (PCH) during early development. We also followed the localization of EZH2 and BEND3, previously shown in ESCs to drive PRC2 to hypomethylated PCH. We show that the location of H3K27me3 at PCH, in addition to H3K9me3, is a defining feature of embryonic cells in vivo. Moreover, it may play an important role in structuring PCH and preserving genomic integrity at a time of globally relaxed chromatin. At peri-implantation stages, while DNA methylation is still low, EZH2 and then H3K27me3, leave PCH in epiblast progenitors at the time of their spatial segregation from primitive endoderm cells, while BEND3 remains there up to implantation. The comparison with stem cells (ESCs and TSCs) reveals that the epigenetic marks (i.e. H3K9me3 and H3K27me3) of PCH are reset during in vitro derivation and only partially restored thereafter. This highlights possible divergences between in vitro and “in embryo” epigenetic regulation regarding constitutive heterochromatin.

## Introduction

After fertilization, the newly formed diploid genome of the zygote has to be reprogrammed to erase the gametic epigenetic features and allow the initiation of transcription (reviewed in^1^). Early development is marked by the progressive relocalization of various histone post-translational modifications as well as the establishment of DNA methylation^2–4^. Along with this epigenomic reorganization, the first lineage commitment takes place a few days after fertilization when embryonic cells within the Inner Cell Mass (ICM) segregate from the surrounding extra-embryonic Trophoblast (TE). The ICM will later divide into the pluripotent Epiblast (EPI) and the Primitive Endoderm (PrE)^5^. In the mouse embryo, pluripotency is not a steady state but rather an “in vivo continuum” from early blastocyst (E3.0) to gastrulation stages (E7.5)^6^. Based on the differential characteristics of pluripotent cells observed along with embryonic development, previous studies defined different states of pluripotency from naive to primed^7^. Naive pluripotency is reached in the ICM or in the pre-implantation epiblast while primed pluripotency characterizes the post-implantation pluripotent states that exist in vivo*.* They both can be caught up and maintained in vitro in specific culture media^7^. Embryonic Stem Cells (ESCs) are representative of the naive pre-implantation ICM/epiblast while Epiblast Stem Cells (EpiSCs) are equivalent to the post-implantation epiblast. Similarly, Trophoblast Stem Cells (TSCs) can be obtained from pre-implantation trophoblast or post-implantation Extraembryonic Ectoderm^8^. Naive and primed pluripotent states differ from each other as they respond to distinct signalling pathways, express different pluripotency markers, and exhibit different epigenetic features and chromatin structure^6,9^. The chromatin is more relaxed in the pre-implantation pluripotent cells than in the post-implantation ones or in other extraembryonic lineages, these specificities are conserved in their in vitro counterparts^9^. Recent studies have shown distinctive chromatin-associated proteome in ESCs according to their culture medium, notably at pericentromeric heterochromatin^10,11^.

In differentiated cells, pericentromeric sequences (called major satellites in mice) are part of the heterochromatin compartment and maintained under transcriptional repression^12^. Epigenetic features such as strong DNA methylation and specific enrichment of H3K9me3 support the transcriptional control of major satellites in somatic cells^13^. These sequences are packed in clusters of condensed chromatin called chromocenters, forming foci heavily stained by the nuclear dye DAPI^14^. During mouse development, the formation of chromocenters at the late 2-cell stage requires a mandatory transcription burst of major satellite sequences, which are then maintained at a low level of transcriptional activity^15,16^. Two studies have shown that chromocenters of ESCs are partially marked by H3K27me3, even though H3K27me3 is normally found at facultative heterochromatin^10,11^. Such uncommon localization of H3K27me3 at pericentromeric heterochromatin (PCH) only occurs when ESCs are cultured under the naïve ground state condition, i.e. with MEK and Gsk3 inhibitors (2i/LIF). When ESCs are cultured in serum/LIF-based medium, enrichment of H3K27me3 at PCH was reported only upon the absence of H3K9me3 or DNA methylation in mutant cells^17,18^. In such mutant cells, a cross-talk between a methyl-sensible DNA binding protein BEND3, PRC1 and PRC2 is at play to establish H3K27me3 at PCH.

As H3K27me3 exhibits differential enrichment at chromocenters between naïve and primed pluripotent states (ESCs in 2i/LIF vs. EpiSCs), we wondered whether the presence of H3K27me3 at chromocenters was also dynamically regulated during mouse early development. We provide a precise overview of the localization of H3K27me3 at chromocenters during the transition from pre- to post-implantation stages in each lineage, and we show that H3K27me3 is differently regulated in the pluripotent cells in comparison to extra-embryonic lineages. We also examined the presence of BEND3 and of EZH2, the enzyme in PRC2 responsible for the tri-methylation of H3K27, at chromocenters. In addition, we show that major satellite sequences are transcribed at early blastocyst stages and then repressed in the post-implantation embryo. We highlight that the non-canonical H3K27me3 enrichment is a physiological feature of chromocenters during pre-implantation development. However, the presence of H3K27me3 at chromocenters seems not to be involved in the transcriptional regulation of major satellite sequences. We also demonstrate that the epigenetic dynamics of PCH differ in vivo from what is observed in vitro, suggesting that current cell culture conditions fail to preserve the native epigenetic characteristic of embryonic chromocenters.

## Results

### H3K27me3 progressively accumulates at chromocenters up to the 16-cell stage

As non-classical labelling of chromocenters by H3K27me3 at chromocenters was observed among a fraction of naïve pluripotent cells in vitro^11^, we aimed at investigating its presence in vivo in the corresponding cells of embryos by immunostaining. Embryos from the 2-cell stage onwards were used to trace back this mark as early as the formation of chromocenters. H3K9me3 was also stained as the hallmark of heterochromatin. DAPI counterstaining was used to delimit chromocenters as they are mostly composed of clustered pericentromeric heterochromatin which, based on their A-T rich composition, are visible as DAPI-dense foci in the interphase nucleus^19^.

**Figure 1:**
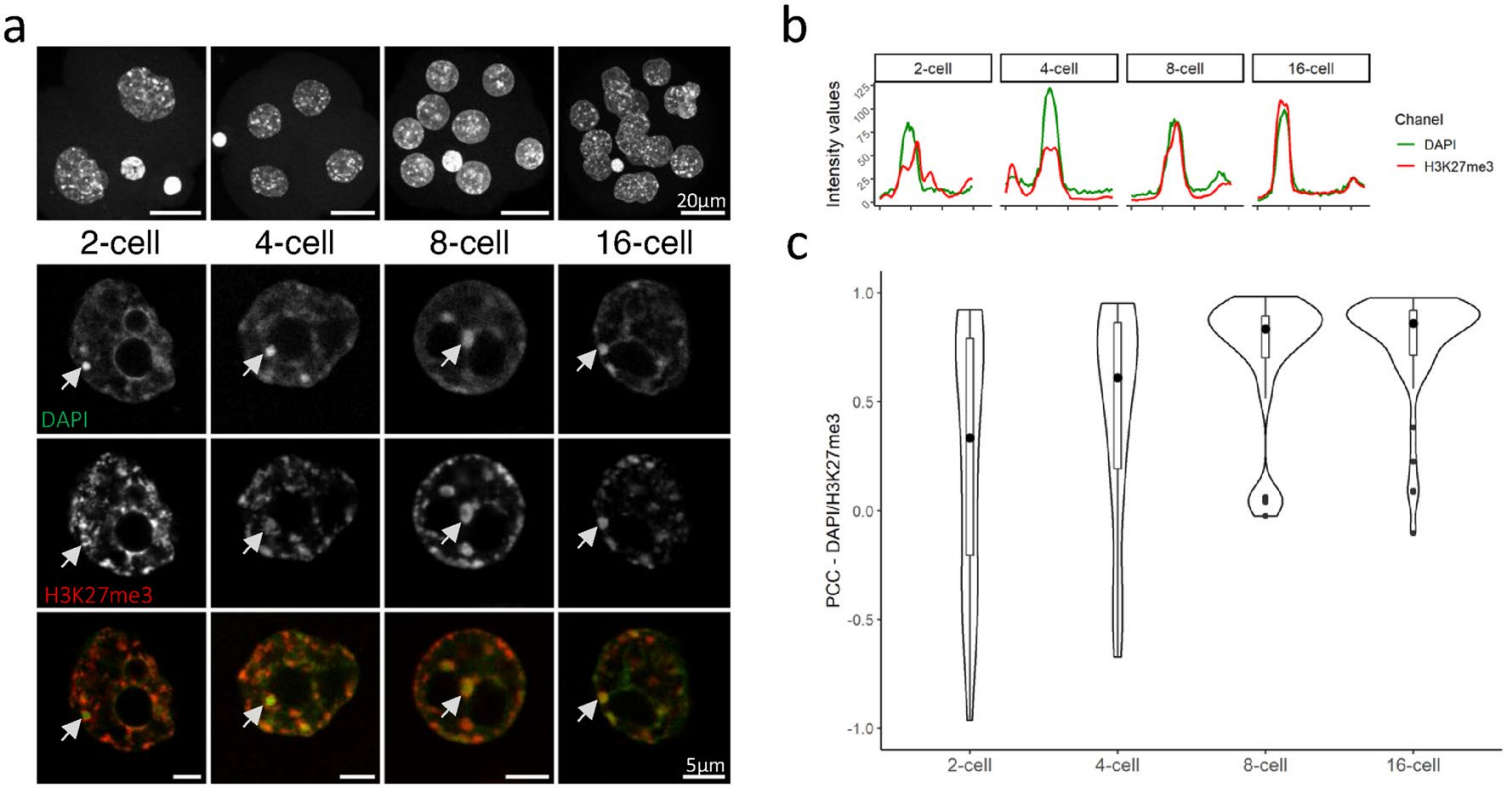
Pattern of H3K27me3 in 2-cell to 16-cell embryos. (**a**) Upper panel row shows the Z-projection of a whole embryo, counterstained with DAPI. Scale bar represents 20 µm. Bottom panels show a single section of a representative nucleus for each stage after staining with DAPI (green) and H3K27me3 (red). Last row is the merge of the two signals. Scale bar represents 5 µm for zoomed-in nuclei. (**b**) The graphs show the intensity profiles of DAPI (green) and H3K27me3 (red) signals across a single chromocenter highlighted by an arrowhead in (**a**). (**c**) Violin plots show the distribution of the Pearson’s Correlation Coefficients (PCC) between DAPI (green) and H3K27me3 (red) profiles at chromocenters for each stage (149 chromocenters analysed). Graphs were generated using ggplot2^56^ (v3.3.5) in R and figures were arranged with FigureJ^55^ in Fiji^54^ (v1.53c).

Embryos, with co- immunostaining of both H3K27me3 and H3K9me3, from 2-cell to 16-cell stages were first analysed (Fig. 1a and Supplementary Fig. 1a). H3K9me3 was enriched at all chromocenters and overlaid with DAPI, in all blastomeres, and at all stages (Supplementary Fig. 1a) while H3K27me3 displayed a more dynamic pattern (Fig. 1a). In the 2-cell stage embryo, H3K27me3 was already located at most chromocenters (about 80%, data not shown) and formed cloudy staining encompassing DAPI-dense foci (arrow in Fig. 1a). A representative cross-sectional view of H3K27me3 intensity profile (red line in Fig. 1b) showed that the signal is accumulated in and around the DAPI intensity peak corresponding to the chromocenter (green line in Fig. 1b).

At later cleavage stages, H3K27me3 progressively accumulated at chromocenters as shown by the progressive overlay of both H3K27me3 and DAPI staining at DAPI-dense foci in the 4-cell and then the 8-cell stage embryos (Fig. 1a). Similarly, the shape of H3K27me3 fluorescent intensity profile increasingly resembled that of DAPI (Fig. 1b). In the 16-cell stage embryos, chromocenters were fully enriched with H3K27me3 (Fig. 1a), as they are with H3K9me3—which fluorescent intensity profile matches that of DAPI at all stages—(Supplementary Fig. 1b).

The increasing similarity of the fluorescent intensity profiles of DAPI and H3K27me3 across stages reflected the progressive accumulation of this histone modification at chromocenters. We extended this analysis by measuring the similarity between DAPI and H3K27me3 (Fig. 1c) or H3K9me3 (Supplementary Fig. 1c) intensity profiles across chromocenters and computed the Pearson’s Correlation Coefficients (PCC) between pairs of profiles (see Methods for further details). As expected, the PCC corresponding to the accumulation of H3K9me3 fluorescent signal at DAPI-dense foci were stable and close to 1 (Supplementary Fig. 1c; median 0.90), while regarding H3K27me3, they were more variable and increased progressively (0.33 to 0.89) up to the 16-cell stage (Fig. 1c). This dynamic and non-canonical localization of H3K27me3 at chromocenters observed by immunostaining was confirmed using published H3K27me3 ChIP-Seq data from Liu and collegues^20^, showing that major satellites sequences were indeed increasingly enriched by H3K27me3 during pre-implantation development (Supplementary Fig. 1d). Hence, by the 16-cell stage, H3K27me3 and H3K9me3 similarly accumulate at all chromocenters.

### H3K27me3 exhibits three different patterns in the peri-implantation embryo

**Figure 2:**
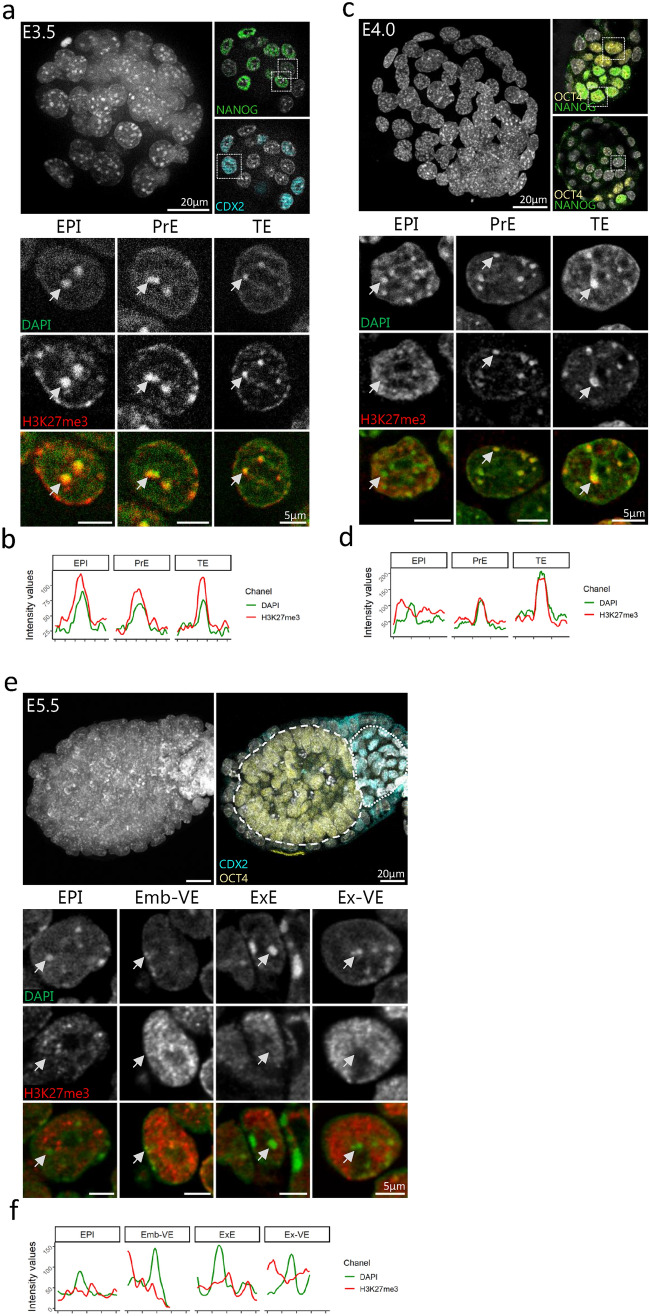
Comparison of the pattern of H3K27me3 at chromocenters between embryonic to extra-embryonic lineages in peri-implantation stages embryos. (**a, c** and **e**) Upper left panel shows the Z-projection of a whole E3.5 (**a**), E4.0 (**c**) or E5.5 (**e**) embryo, counterstained with DAPI. Scale bar represents 20 µm. Upper right panels show a section with a highlight of ICM/EPI cells stained with NANOG (**a**, **c**) or OCT4 (**c**, **e**), TE or ExE cells stained with CDX2 (**a**, **e**) or PrE/VE cells stained with OCT4 only (**c**). Bottom panels show a representative nucleus for each presumptive lineage after immunostaining with H3K27me3 (red) and DAPI at E3.5 (**a**), E4.0 (**c**) and E5.5 (**e**). The last row is the merge of the two signals. EPI = Epiblast cells, PrE = Primitive Endoderm cells, TE = Trophoblast cells, ExE = Extra-embryonic Ectoderm cells, Emb-VE/Ex-VE = Visceral Endoderm cells in embryonic/extra-embryonic region respectively. (**b**, **d** and **f**) Graphs show the intensity profiles of DAPI (green) and H3K27me3 (red) signals across a single chromocenter highlighted by arrowheads in lower panels (**a**, **c** and **e)**. Graphs were generated using ggplot2^56^ (v3.3.5) in R and figures were arranged with FigureJ^55^ in Fiji^54^ (v1.53c).

Next, we analysed the enrichment of H3K27me3 at chromocenters at further stages from young cavitating blastocyst (E3.25) to post-implantation embryo (E5.5), to cover the transition from the emergence of naïve pluripotency to the onset of primed pluripotency (Supplementary Fig. 2a). At peri-implantation stages, three distinct patterns of H3K27me3 can be observed, depending on the lineage (embryonic or extra-embryonic) and the implantation status. We choose to highlight the three stages that are the more representative of the various patterns of H3K27me3 i.e. at E3.5, E4.0, and E5.5 days of development (Fig. 2).

The accumulation of H3K27me3 at chromocenters described at the 16-cell stage was maintained during the first lineage specification in conjunction with the onset of cavitation in the mouse embryo, from E3.25 to E3.5 stages (Fig. 2a and Supplementary Fig. 2b). At E3.5, NANOG positive epiblast precursors within the Inner Cell Mass (ICM, NANOG positive cells) were mixed with primitive endoderm (PrE) precursors in a “salt and pepper” pattern^5^ while being surrounded by trophectoderm cells (TE) identified by CDX2 (Fig. 2a, upper right panels). No noticeable differences between lineages were observed (Fig. 2a, lower panel, and b).

At the 3.75 stage, the fluorescent intensity profile of H3K27me3 seems to become more uniform in EPI cells compared to extra-embryonic cells, concomitantly with the spatial segregation of EPI and PrE cells within the ICM (Supplementary Fig. 2e). At E4.0, OCT4 is found in both PrE and EPI cells while NANOG is restricted to the pluripotent epiblast (Fig. 2c). Remarkably, the most dramatic change in the pattern of H3K27me3 was observed in EPI cells. While H3K27me3 remained accumulated at chromocenters in both TE and PrE cells, it converted into a diffuse and granulated signal in EPI cells (Fig. 2c), with less specific accumulation at DAPI-dense foci compared to that observed at TE and PrE chromocenters (Fig. 2d). At post-implantation stages (E5.5), H3K27me3 was no more found at chromocenters, in all cells of the embryo (Fig. 2e, f). At all these stages, the staining profile of H3K9me3 remained unchanged i.e. being still enriched at chromocenters and overlapping with H3K27me3 where this mark was still present (Supplementary Fig. 2b–j).

**Figure 3:**
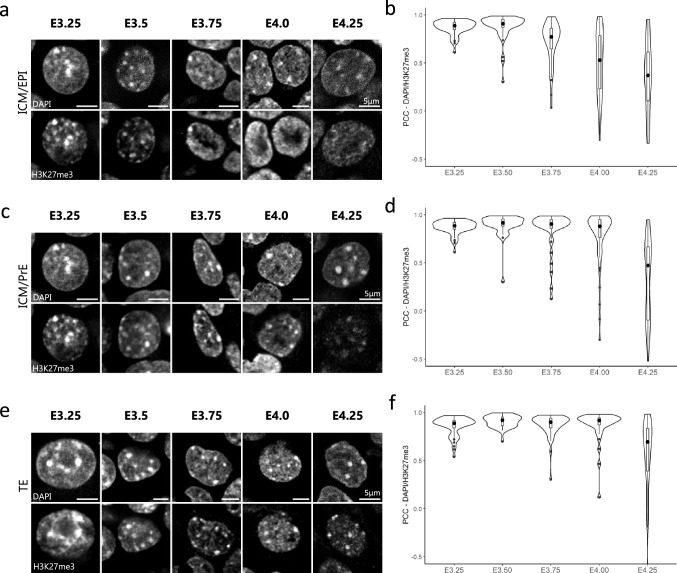
Correlation dynamics between DAPI and H3K27me3 signals at chromocenters at blastocyst stages. **(a**, **c** and **e**) Pattern of H3K27me3 in a representative nucleus of EPI (**a**), PrE (**c**) and TE (**e**) cells at different blastocyst stages (from E3.25 to E4.25). DNA is stained with DAPI. Scale bar represents 5 µm. (**b**, **d** and **f**) Violin plots show the distribution dynamics of the Pearson’s Correlation Coefficients (PCC) between DAPI and H3K27me3 profiles at chromocenters in EPI (**b**), PrE (**d**) and TE (**f**) cells at various stages (262, 223 and 196 chromocenters analysed respectively). Graphs were generated using ggplot2^56^ (v3.3.5) in R and figures were arranged with FigureJ^55^ in Fiji^54^ (v1.53c).

The decrease of H3K27me3 accumulation at chromocenters—noticeable in staining experiments—during the regionalization of the pluripotent epiblast was corroborated by the decrease in the PCC between DAPI and H3K27me3 intensity plots at chromocenters in EPI cells (Fig. 3a,b). Only at E4.25, PrE and TE cells started to exhibit heterogeneity regarding the accumulation of H3K27me3 at chromocenters, with some cells resembling EPI cells while others maintain the H3K27me3 enrichment at chromocenters (Fig. 3c–f). At the time of embryo implantation (E4.5 and beyond), H3K27me3 became diffuse in all cells of the three lineages, similar to that observed in post-implantation embryos (Fig. 2e). In the course of the changes in H3K27me3 pattern, the persistence of stable accumulation of H3K9me3 at all DAPI-dense foci ruled out the disappearance of chromocenters, corroborated by stable PCC (Supplementary Fig. 3).

### The profile of major satellite transcription is variable during peri-implantation development

Because H3K27me3 exhibits differential enrichment at chromocenters depending on either the stage or the lineage, we asked whether the transcription status of major satellites was modulated according to the presence of H3K27me3 at chromocenters. To this aim, we performed immuno-RNA-FISH to detect simultaneously the NANOG positive pluripotent cells and the accumulation of major satellite transcripts. After the well-known initial burst of transcription at 2-cell stage^21,22^, a low level of transcription of major satellite was maintained until the blastocyst stage as 96% of cells from 4-cell to 16-cell stages exhibited no more than 2 foci (Supplementary Fig. 4a,b).

**Figure 4:**
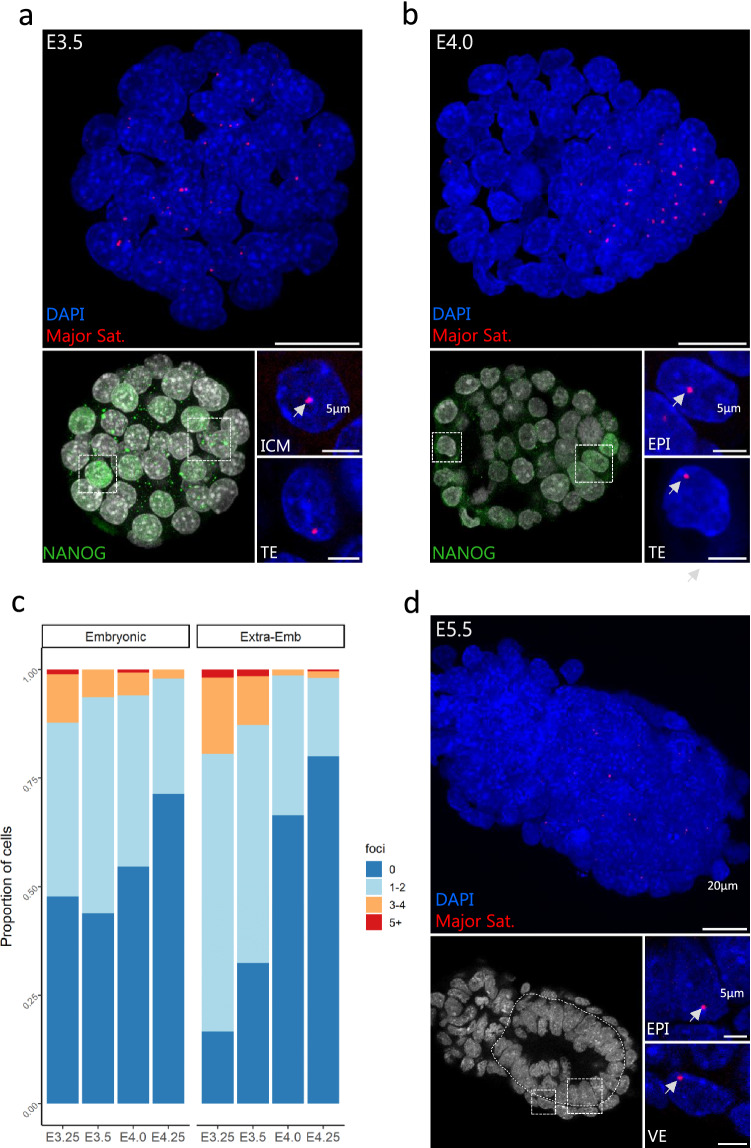
Transcription dynamics of major satellites in peri-implantation embryos assessed by RNA-FISH. (**a**, **b**) left panels show the Z-projection of whole embryos processed for immuno-RNA-FISH at E3.5 (**a**) and E4.0 (**b**). Scale bar represents 20 µm. EPI cells are either identified with NANOG staining (lower left panels, **a** and **b**) or morphologically (**c**). Lower right panels show zoomed-in nuclei of both embryonic or extra-embryonic lineages, with DAPI (blue) and major satellite (red) signals. Scale bar is 5 µm. **(d)** Proportion of cells in EPI or Extra-embryonic lineages exhibiting 0, 1–2, 3–4 and 5 or more (5 +) RNA-FISH foci per cell at the different indicated blastocyst stages (3400 nuclei analysed). Graphs were generated using ggplot2^56^ (v3.3.5) in R and figures were arranged with FigureJ^55^ in Fiji^54^ (v1.53c).

At blastocyst stages (E3.25 to E4.25), both embryonic and extra-embryonic lineages showed higher heterogeneity regarding the number of foci per nucleus and the proportion of cells transcribing major satellite (Fig. 4a–c). Initially, extra-embryonic cells displayed more cells with active foci (83%) than epiblast cells but prior to implantation at E4.25, the proportion of transcribing cells decreased to less than 20% in both lineages (Fig. 4c).^.^At post-implantation stages, very few cells still exhibited transcription foci (Fig. 4d). Of note, the sharp decrease in the extra-embryonic cells takes place while H3K27me3 is still well enriched at PCH, suggesting a limited role of this mark on the control of satellite transcription.

We then ask whether the reduction of major satellite transcripts could be better correlated with the onset of de novo DNA methylation. Using published dataset from Zhang and colleagues^23^, we reanalysed the 5meC level on major satellite sequences in the ICM, TE and EPI cells at blastocyst and early post-implantation stages (Supplementary Fig. 4c). This shows that de novo DNA methylation starts after E4.0, while the decrease of satellite transcription has already started in both lineages, ruling out a strict correlation of 5meC level and PCH transcriptional activity at blastocyst stages.

### Dynamic of the H3K27me3 writer EZH2 and of BEND3

**Figure 5:**
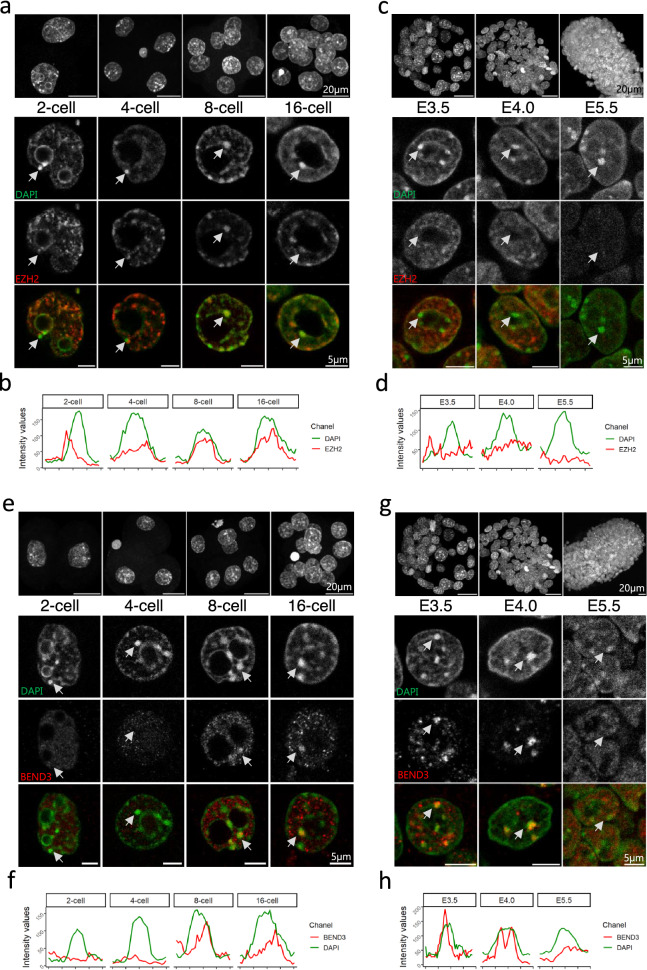
Patterns of EZH2 and BEND3 during peri-implantation development. (**a**, **c**) Upper panels show the Z-projection of a whole embryo at 2-, 4-, 8- and 16-cell (**a**) and at E3.5, E4.0 and E5.5 (**c**). Lower panels show a representative nucleus at each stage after staining with DAPI (green) and EZH2 (red). Arrows point to a chromocenter using to draw the intensity profile shown in (**b**) and (**d**). (**e**, **g**) Upper panels show the Z-projection of a whole embryo at 2-, 4-, 8- and 16-cell (**e**) and at E3.5, E4.0 and E5.5 (**g**). Lower panels show a representative nucleus at each stage after staining with DAPI (green) and BEND3 (red). Arrows point to a chromocenter using to draw the intensity profile shown in (**f**) and (**h**). Graphs were generated using ggplot2^56^ (v3.3.5) in R and figures were arranged with FigureJ^55^ in Fiji^54^ (v1.53c).

To further explore the dynamics of H3K27me3 at PCH, we asked whether its writer, EZH2, followed a similar pattern. We found that EZH2 localized around chromocenters from 2-cell up to 16-cell stage (Fig. 5a,b), concomitantly with chromocenter clustering and H3K27me3 enrichment (Fig. 1). At E3.5, whereas H3K27me3 was still localized at PCH (Fig. 2a), EZH2 became less specifically localized around chromocenters in all cells of the blastocyst (Fig. 5c,d and Supplementary Fig. 5a) and remained diffuse afterwards (Supplementary Fig. 5b,c). Hence, the writer leaves the vicinity of PCH prior to the removal of the histone modification, which will be effective at E4.0 in the epiblast cells (Fig. 3).

We also intended to assess BEND3 location since this protein is critical for the recruitment of PRC2 at PCH in ESCs depleted in DNA methylation^18^. BEND3 is a conserved DNA binding protein that plays a role in heterochromatinization and transcription inhibition^24^. We therefore examined the pattern of BEND3 (Fig. 5e–h). BEND3 started to be enriched at PCH only at 8-cell stage (Fig. 5e,f), so after the recruitment of both EZH2 and H3K27me3 that occur at 2-cell stage (Fig. 5 and Fig. 1). The BEND3 enrichment at PCH was conserved up to E5.0 in all cells, and lost at E5.5 (Fig. 5g,h and Supplementary Fig. 5a–c). In EPI cells, the dynamic of BEND3 at PCH follows quite closely the de novo acquisition of DNA methylation (Supplementary Fig. 4c) but is disconnected from the presence of EZH2, which appears and disappears earlier. Hence, BEND3 is not involved in PRC2 recruitment at PCH in the embryo.

**Figure 6:**
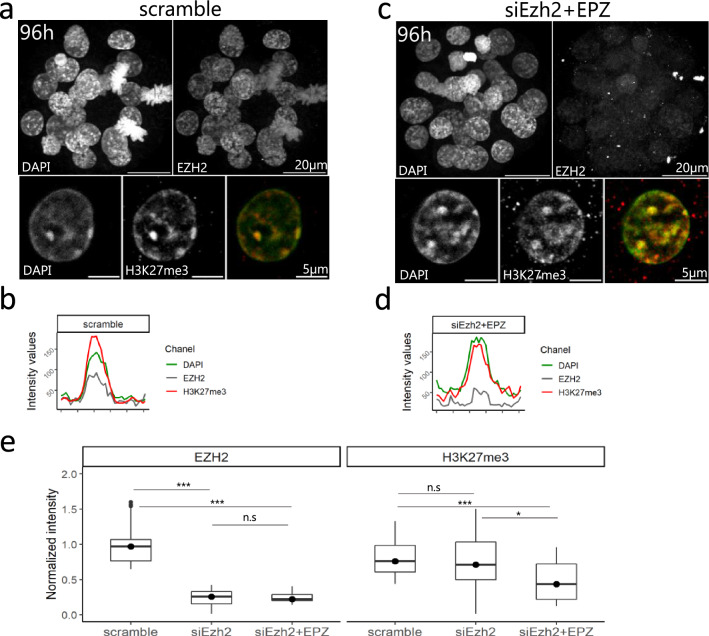
Knock-down of EZH2 and its effects on H3K27me3 accumulation at PCH. (**a**, **c**) Early blastocysts at 96 h of culture after electroporation with scramble (**a**) or siEzh2 (**c**) and stained for EZH2 (grey), DAPI (green) and H3K27me3 (red). Upper panels show a z-projection of a whole embryo, while lower panels show a representative nucleus. (**b**, **d**) The graphs show the intensity profiles of DAPI, H3K27me3 and EZH2 signals across a single chromocenter. (**e**) Quantification of total signal intensity of EZH2 and H3K27me3 in nuclei (n = 20) of scramble, siEzh2 and siEzh2 + EPZ treated embryos. *** Pvalue < 10^−3^; ns = non significant; Mann–Whitney test. Graphs were generated using ggplot2^56^ (v3.3.5) in R and figures were arranged with FigureJ^55^ in Fiji^54^ (v1.53c).

We then aimed at interfering with H3K27me3 recruitment at chromocenters by targeting EZH2 transcripts using RNA interference. Zygotes were electroporated with either siRNA against Ezh2 (siEzh2) or scramble and the pattern of H3K27me3 and EZH2 was assessed with immunostaining after 72 h of in vitro culture. Furthermore, embryos were cultured in the presence of EPZ-6438 (EPZ), an inhibitor of EZH enzymatic activity^25^. EPZ was previously used to remove H3K27me3 in 2i-ESCs^10^ and was used here as a mean to get rid of maternal EZH2 activity^26^. EZH2 was dramatically reduced in siEzh2 treated embryos compared to control (76% reduction of fluorescence intensity, Fig. 6). However, quantification of the total fluorescence signal revealed only a partial reduction of H3K27me3 in siEzh2 (10%) and siEZH2 + EPZ (40%) treated embryos (Fig. 6e). Furthermore, PCH remained enriched for H3K27me3, with no obvious difference between scramble and siEzh2 + EPZ treated embryos (Fig. 6a–d). No difference in major satellite transcription was also observed (Supplementary Fig. 5d).

In female embryos, the imprinted inactive paternal X chromosome accumulates a large amount of H3K27me3 and EZH2 at all pre-implantation stages^27,28^. In siEzh2+EPZ treated embryos, although the inactive X was still recognizable by a faint patch of EZH2 combined with a slight accumulation of DAPI, H3K27me3 was dramatically reduced at this locus (65% reduction of fluorescence intensity, Supplementary Fig. 5e–i). This further indicates that global depletion of EZH2 and impairment of its activity in the embryo affects the constitutive heterochromatin domains in a different manner than the facultative ones.

### Resetting the PCH epigenetic structure during establishment of embryonic stem cells

Our results show that the epigenetic structure at PCH is highly dynamic at blastocyst stage prior to implantation. The abrupt loss of H3K27me3 between E3.5 and E4.0 and the constant presence of H3K9me3 in vivo are not faithfully mirrored in ESCs, even if maintained in the ground state condition where they are transcriptionally close to the early epiblast^10,29^. We therefore asked if PCH was remodelled during ESC derivation.

**Figure 7:**
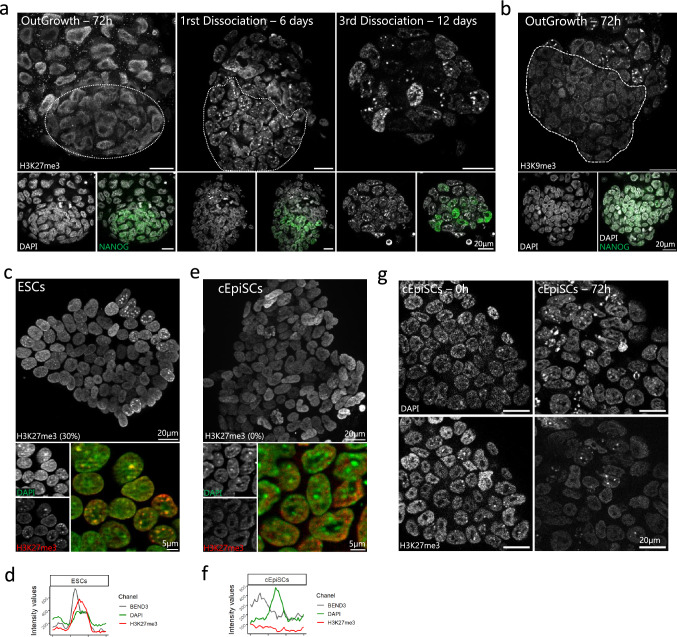
Comparative H3K27me3 enrichment at chromocenters during ESCs derivation and cEpiSCs conversion. (**a**) From left to right: H3K27me3 staining profile at different time points of ESC derivation from blastocysts cultured in 2i/LIF medium. (**b**) H3K9me3 staining in 72 h outgrowth. For (**a**) and (**b**), DAPI and NANOG staining are presented in the lower panels. The pluripotent cell population is delineated by a white dashed line. (**c**, **e**) ESCs and 10 days converted EpiSCs (**e**) colony stained for H3K27me3 (red), DAPI (green) and merge signals. (**d**, **f**) The graphs show the intensity profiles of DAPI, H3K27me3 and BEND3 signals across a single chromocenter for ESCs (**d**) and cEpiSCs (**f**). (**g**) Dynamics of H3K27me3 disappearance at 0 and 3 days post-medium switching from 2i/LIF to FGF2/Activin. Graphs were generated using ggplot2^56^ (v3.3.5) in R and figures were arranged with FigureJ^55^ in Fiji^54^ (v1.53c).

E3.5 blastocysts were cultured in 2i/LIF medium until outgrowth was formed. After 3 days of culture, the initially spotted pattern of H3K27me3 was lost in all cells (NANOG positive and NANOG negative) of the outgrowth, and then reappeared after the first dissociation (day 6), but remained heterogeneous in the colonies (Fig. 7a). At the same time, both H3K9me3 and BEND3 were lost at PCH in 72 h outgrowth, but only in NANOG positive cells (Fig. 7b and Supplementary Fig. 6a). Once established, ESCs maintained an heterogeneous but stable pattern for H3K27me3, with a third of the population exhibiting accumulation of H3K27me3 at PCH (Fig. 7c,d), in agreement with previous results^10^. When cells were converted to primed cEpiSCs in the presence of FGF2 and Activin, they exhibited a diffuse pattern for H3K27me3, as expected (Fig. 7e,f). On the other hand, BEND3 was reacquired at PCH in about half the ESC population (Supplementary Figs. 6b and 7d) and then lost in converted EpiSCs (Supplementary Figs. 6c and 7f). The pattern of H3K27me3 was also examined at the intermediate EpiLC stage, after 3 days of conversion (Supplementary Fig. 6 h). Intriguingly, cells retaining H3K27me3 at chromocenters were still present in the culture (Fig. 7g), meaning the kinetic of H3K27me3 loss at chromocenters was much slower and heterogeneous in vitro than in vivo*.*

To further compare pluripotent cells in vitro and in vivo, major satellite transcription was also assessed by RNA-FISH. Interestingly, the proportion of ESCs exhibiting RNA-FISH foci (66%) was close to that observed in the ICM of the young blastocyst (E3.25/E3.5—approx. 50%), while most EpiSCs did not transcribe major satellite, as in primed epiblast (Supplementary Fig. 6d).

At last, H3K27me3, BEND3 and major satellite expression were assessed in TSCs as an in vitro surrogate of TE. TSCs were cultured in the defined serum-free medium (FAXY^30^), which reduces the propensity of serum-cultured TSCs to spontaneously differentiate. No accumulation of H3K27me3 was observed at chromocenters, which were enriched in BEND3 in 41% of the cells, suggesting these TSCs were representative of an intermediate state between TE and ExE (Supplementary Fig. 6e–g). However, the proportion of cells that exhibit foci of major satellite transcripts (44%) was more in line with the transcription status of pre-implantation TE than post-implantation ExE (Supplementary Fig. 6d).

Altogether, our results show that in vitro derivation of stem cells from pre-implantation blastocyst (ESCs and TSCs) does not faithfully preserve the epigenetic features at PCH in embryo, highlighting the importance of in vivo studies as a complement of the in vitro use of stem cells.

## Discussion

In the present study, we describe the dynamics of the epigenetic profile of the chromocenters in the mouse embryo, from the compaction of pericentromeric heterochromatin (PCH) at the 2-cell stage, through the establishment of the first lineages, up to the primed pluripotency state in early post-implantation embryo (supplementary Fig. 7). We highlight here that the location of H3K27me3 at PCH, in addition to the hallmark of heterochromatin H3K9me3, is in fact a defining feature of embryonic cells in vivo, regardless of the lineage (embryonic or extra-embryonic). The writer EZH2 and H3K27me3 are both enriched around chromocenters already at 2/4-cell stage, then within chromocenters at 8-cell stage. At this stage, BEND3 is also present around this region. At peri-implantation stages, our results unravel a finely orchestrated choreography of H3K27me3, EZH2 and BEND3 that takes place at PCH between E3.0 and E4.0, while DNA methylation is still low (Supplementary Fig. 7). EZH2 leaves PCH just before the histone modification, while BEND3 remains up to the rise of de novo methylation. Interestingly, H3K27me3 loss at PCH happens concomitantly to the spatial segregation of epiblast from PrE, making it a reliable marker of the onset of epiblast maturation.

In addition to H3K9me3, strong DNA methylation is involved in the maintenance of a repressive epigenetic environment at PCH in differentiated cells^13^. However, global genome demethylation is one of the many rearrangements that accompany fertilization and early development^31^. In our study, we show that the progressive colonization of chromocenters by H3K27me3 coincides with the progressive demethylation of the genome, including major satellite sequences^32–34^. In ESCs as well, upon depletion of DNA methylation, H3K27me3 progressively accumulates at PCH^17^. This is also the case when ESCs are cultured in 2i/LIF medium that maintains them in a state closer to the pre-implantation ICM^10,11^, but H3K27me3 staining remains heterogeneous^10^. Hence, the homogeneous coexistence of H3K27me3 with H3K9me3 and reduced DNA methylation is a feature of pre-implantation embryo (this study).

PCH is asymmetrically marked by H3K9me3 (maternal) or H2AK119Ub/H3K27me3 (paternal) following fertilization^35,36^, suggesting that the presence of H3K27me3 at chromocenters in the 2-cell embryo is partly inherited from parental genomes. Recent data reporting crosstalk between PRC1 and PRC2 complexes and their associated histone modifications (H2AK119Ub and H3K27me3, respectively)^37,38^ suggest that the presence of H2AK119Ub or the action of a variant PRC1 complex would be necessary for the apposition of H3K27me3 during mouse early cleavage stages. At 8-cell stage, the lower level of DNA methylation appears sufficient to allow binding of BEND3 around chromocenters. EZH2 being already present, BEND3 does not drive EZH2 at PCH, as it does *in vitro* in *Suv39h*dn or *Dnmt*TKO ESC^18^. However, it may help to maintain and/or stabilize H3K27me3. In the context of embryonic development, we thus hypothesize that PRC1/H2AK119Ub and BEND3 are successively involved in maintaining H3K27me3 at chromocenters.

We observed that the depletion of EZH2 (and inhibition of its activity) did not significantly affect H3K27me3 enrichment at PCH. EZH2 is present maternally in the zygote, allowing the presence of H3K27me3 on the paternal pronucleus^35^ and this maternal form is mainly responsible for the presence of this mark up to early blastocyst stage^39^. Hence it is possible that a low level of enzyme is sufficient to maintain the modification over cell division, highlighting the stability of H3K27me3 at PCH.

Such a robust and resilient system is in favour of an important role in the preservation of heterochromatin integrity. During preimplantation development, the chromatin is immature and largely decondensed. The progressive loss of DNA methylation also requires other mechanisms to preserve genomic integrity at repeat regions such as PCH. Although the variations in transcriptional activity at major satellites appears uncoupled to the presence of H3K27me3, especially at late blastocyst stage, it is still possible that H3K27me3 together with H3K9me3 could play a role in structuring PCH and preserving genomic integrity in a context of globally relaxed chromatin. A growing body of studies shows that a certain level of transcriptional activity is important for the patterning and maintenance of the integrity of heterochromatin^40,41^. This is supported by the existence of motifs recognized by transcription factors within PCH^42^. On one hand, recent data suggest that H3K9me3 in early mouse embryos may not be as repressive as it is in differentiated cells, as embryonic knock-down of its writer *Suv39h1* exhibits no drastic increase in major satellite transcription^43^. On the other hand, H3K27me3 may exert a milder repression of repeat elements than H3K9me3, thus compatible with maintenance of a low transcription rate^44,45^. Such low level of transcripts seems to be important as loss of PCH transcripts at 2-cell stage impairs chromocenters clustering and further development^21^, and, in ESCs, loss of PCH transcription induces the condensation of heterochromatin that generates genomic instability and mitotic defects^46^.

At peri-implantation stages, EZH2 then H3K27me3 leave PCH upon spatial segregation of epiblast cells. As this happens in less than one cell division, we assume that H3K27me3 withdrawal is an active phenomenon, independent of the restoration of DNA methylation that has not yet started. BEND3 remains for another day at chromocenters (E5.0). A very recent study has highlighted a role for BEND3 during early gastrulation as a gatekeeper of bivalent gene silencing^47^. We propose that the accumulation of BEND3 at PCH up to the early post-implantation stage could serve as reservoir for future usage elsewhere in the genome, at a period of extensive relocalization of H3K27me3, especially at bivalent promoters^1^.

We show that in E3.5 embryos explanted in vitro in 2i/LIF medium, both H3K27me3 and H3K9me3 disappear from chromocenters during the early steps of derivation before being heterogeneously re-established during stabilization of ESCs. In addition, EZH2 is not re-established at PCH^10^ whereas BEND3 heterogeneously is. Furthermore, the presence of BEND3 at PCH is not related to H3K27me3-marked chromocenters, both in ESCs and in TSCs, insinuating BEND3 has a role other than inducing PRC2 recruitment to PCH. This transient loss and re-establishment of both H3K27me3 and H3K9me3 at the chromocenters suggests a “reset” of the epigenetic profile of PCH in ESCs. Indeed, it has been described that the transcriptome of ES-derived epiblast was partially altered during this process^48^. Intriguingly, the reset is incomplete as the H3K27me3 and H3K9me3 pattern at PCH is not re-established in all cells (this study and^10^). In addition, the removal of H3K27me3 from PCH during the in vitro conversion of ESC to the primed state takes more than 72 h to happen (this study and^49^) which is in sharp contrast with the fast remodelling of H3K27me3 observed *in vivo* upon epiblast progression toward the primed state. This suggests a passive erasure in vitro in contrast to an active one in vivo. During differentiation of ESCs, the pattern of H3K27me3 is reshuffled and this is in agreement with passive erasure^50^ because its happens even after depletion of the two known H3K27me3 erasers, KDM6A/B.

Altogether, these results suggest that a different regulatory epigenetic network acts at chromocenters and maybe elsewhere in the genome as well, in naive pluripotent cells in vitro compared to their in vivo counterparts. Importantly, our study highlights possible divergences between in vitro and “in embryo” epigenetic regulation, at least concerning constitutive heterochromatin.

## Methods

### Ethics statement

Animal procedures were carried out according to French national rules on Ethics and Animal Welfare in the Animal Facility. This work was approved by the French Ministry of Higher Education, Research, and Innovation (n° 15–55 and 21–01) and the local Ethical Committee (INRAE Jouy-en-Josas Centre). The study was carried out in compliance with the ARRIVE guidelines.

### Embryos collection and pre-treatment

Embryos were obtained at various stages by natural mating of CD1/CD1 mice. Pre-implantation blastocysts were collected by uteri flushing with pre-heated M2 medium (Sigma) at 90 h (E3.25), 95–97 h (E3.5/E3.75), 100–102 h (E3.75/E4.0) and at 107–109 h (E4.25/E4.5) post-natural mating. Post-implantation embryos were collected 6 days (E5.5) or seven days (E6.5) post-natural mating by dissection and cleaning in homemade Flushing Handling Medium. For further treatment, zona-pellucida from pre-implantation blastocysts were fragilized by 15 s incubation in acidic tyrode (Sigma) to perform immuno-RNA-FISH experiments. For immunostaining experiments, all embryos were fixed with 2% PFA in PBS (Electron Microscopy Sciences) for 20 min at room temperature. For FISH experiments, embryos were fixed with a solution containing 4% PFA and 0.5% Triton X-100 (Sigma) in PBS at 37 °C for 15 min.

### siRNA experiments

1-cell embryos were collected 24 h after hCG injection from superovulated F1 (C57 × CBA) mice. They were electroporated on a NEPA21 type II electroporator (NEPA GENE Co. Ltd., Japan) following published parameters^51^. Twenty to twenty-five 1-cell embryos were electroporated with 5µg pre-designed siRNA (Ambion™ Invitrogen) diluted in OptiMEM (Sigma). Impedance was maintained between 0.20 to 0.35 kΩ. Treated embryos were then cultured in KSOM medium (Sigma) and fixed at young blastocyst stage (96h–phCG) before further treatment (Immunostaining or RNA-FISH experiments). siRNA targeting Ezh2 (Ambion™ Silencer™ EZH2—ref : AM16708, ID 157427) transcripts were used. Control to siRNA experiments was performed using Ambion™ Silencer™ Negative Control—ref : AM4621 (defined as “scramble” in the study). To reinforce the effect of loss of EZH2 transcripts, embryos electroporated with siEzh2 were cultured in KSOM supplemented with EZH2 inhibitor EPZ-6438 (HY-13803, MedChem Express) at 5µM, renewed daily until embryo fixation.

### Immunostaining

After a short rinse in PBS, embryos were permeabilized at 37 °C in 1% Triton X-100 (Sigma) in PBS for 1 h for blastocysts or 1 h 30 for post-implantation embryos. All embryos were incubated in blocking solution containing 2% BSA in PBS for at least 30 min at room temperature before incubation in primary antibodies diluted in 2% BSA in PBS at 4 °C overnight. After three rinses in PBS, embryos were incubated with secondary antibodies diluted in 2% BSA in PBS for at least 1 h at room temperature. The antibody solution was then rinsed with PBS at least three times before DAPI-counterstaining performed for post-implantation embryos. Embryos were shortly post-fixated in a 2% PFA in PBS solution for 10min at room temperature. All embryos were finally mounted in Vectashield (Vector Laboratories) containing 1/300 DAPI. The antibodies used are specified in Supplementary Table S2.

### Immuno RNA-FISH

Zona-pellucida of early blastocysts was removed using a 0.1N HCl solution. The immunostaining part was performed for pre-implantation embryos as previously described, except for the permeabilization step who was reduced to 30 min in 0.5% Triton X-100 in PBS. After a short rinse step in PBS, embryos were permeabilized in 0.5% Triton X-100 in PBS for 45min for blastocysts or 1h in 1% Triton X-100 for post-implantation embryos. After two rinses in PBS, embryos were incubated in the pre-hybridization solution containing 50% formamide and hybridization buffer for 30 min at 50 °C. Embryos were then put in hybridization solution (containing 50% formamide, 1X hybridization buffer, and LNA probes targeting major satellite RNA—previously denatured at 85 °C for 10 min) at 38 °C overnight. At the end of the hybridization step, embryos were rinsed twice in 2X Saline-Sodium Citrate in water solution before a short DAPI-counterstaining step at 37 °C for 15 min. All embryos were finally mounted in Vectashield (Vector Laboratories) containing 1/300 DAPI.

### ESC derivation from CD1 embryos

CD1 blastocysts were individually cultured on feeders in a 2i/LIF medium until attachment at 37 °C, 5% CO2. Once blastocysts properly attached to the plate, the medium was renewed and the inner cell mass was let to proliferate at least 48 h. The pluripotent cell mass was then isolated and dissociated by a short incubation in TrypLE for 10 min at room temperature. Dissociated cells from each cell mass were then cultured on feeders in the 2i/LIF medium, which was renewed until the first colonies appeared. Grown cell colonies were then dissociated by a short incubation in trypsin 10 min at 37 °C followed by vigorous pipetting. Individualized cells were seeded back on feeders in a 2i/LIF medium for 48 h. Colonies were dissociated a second time with trypsin and put back in culture on feeders before the last dissociation step. ES cells were finally seeded on laminin in Falcon® plates and cultured as previously described^52^.

### Cell culture

ESCs were maintained on laminin in Falcon® plates and cultured in Chemically Defined Medium (CDM) supplemented with 0.7 µM PD0325901 (AxonMedCHem), 2.5 µM CHIR99201 (AxonMedChem), and 700 U/ml LIF (Cell Guidance Systems). Passage of ESCs is performed every 3 days after Trypsin treatment. The conversion of ESCs into cEpiSCs was performed by switching medium to CDM supplemented with 20 ng/ml Activin A (Cell Guidance Systems) and 12 ng/ml FGF2 (Cell Guidance Systems)^53^. cEpiSCs were seeded on serum-coated Falcon® plates and passed every 3 or 4 days after collagenase treatment. TSCs were cultured on serum-coated Falcon® plates according to Ohinata and Tsukiyama^30^ with some modifications: We used a serum-free medium i.e. CDM supplemented with 12 ng/ml FGF2, 20 ng/ml Activin A, 10 nM XAV939 (Sigma) and 5 nM Y27632 (Cell Guidance Systems) called FAXY medium and passed every 3 days. Immunostaining and RNA-FISH experiments were performed on cells cultured at least for 14 days or 10 days for cEpiSCs.

### RNA extraction and RT-qPCR

Total RNA was extracted using RNeasy mini kit (Qiagen) including a DNAse treatment. 500 ng of RNA was used for reverse transcription using Random primers (Invitrogen) and Superscript III (Invitrogen). Quantitative PCR was carried out in triplicates using Kapa Sybr fast mix (Sigma) on a StepOne Plus thermal cycler (Applied Biosystem). Data were normalized using the geometric mean of Sdha and Pbgd using Qbase software (Biogazelle). The primers are described in Supplementary Table S1.

### Acquisition and image analysis

Embryos were observed with a Zeiss LSM 700 confocal microscope (8 bits pixel depth) or a Zeiss Axiovert Apotome microscope (16 bits pixel depth). Early blastocysts were scanned with an ×63 oil-immersed lens (N.A.1.4) and older embryos with an ×40 oil-immersed lens (N.A.1.3). Stack images were acquired with a z-step of 0.5 µm and a frame size of 512 × 512 for small embryos to 2048 × 2048 for the biggest embryos. Cells were observed with a Zeiss Axiovert Apotome microscope (16 bits pixel depth) through an ×63 oil-immersed lens (N.A.1.4). LED wavelengths of 365 nm, 470 nm, 555 nm, and 625 nm (Zeiss Apotome microscope) or LASER wavelengths of 405 nm, 470 nm, 555 nm, and 639 nm (Zeiss confocal microscope) were used for DAPI, Alexa488 or FITC, Cy3, and Cy5 fluorescent staining, respectively.

Images were analysed with the Fiji software^54^. The profiles of fluorescence intensity for DAPI, H3K9me3, and H3K27me3 signals were measured across a chromocenter midline. Chromocenter delineation was done based on the fluorescence intensity profile of DAPI. The intensity profile of a chromocenter corresponds to a bell-shaped curve with values higher than the average fluorescence value of the plot. At the delineated chromocenter, the Pearson’s Correlation Coefficient (PCC) was calculated between the DAPI intensity values and those of H3K27me3 or H3K9me3. The more the fluorescence intensity profile of H3K27me3 or H3K9me3 is similar to the DAPI intensity profile, the closer PCC is to 1. These measures formalize the accumulation of H3K27me3 or H3K9me3 signals at DAPI-dense foci and thus assess the enrichment of H3K27me3 or H3K9me3 at chromocenters.

The signal intensity measurement was performed as follows: for at least 20 nuclei per condition, the total intensity of DAPI, BEND3, EZH2 or H3K27me3 was measured in the entire nuclear volume, including background subtraction. The intensity measurements for BEND3, EZH2 and H3K27me3 were normalized to that of DAPI. For measurements at the inactive X, same process was followed and EZH2 staining was used to delineate the inactive X.

Statistical analysis were performed with R software (v4.1.0, https://www.R-project.org/). Figures were made with Fiji software^54^ (v1.53c) using FigureJ plugin^55^, except for graphs generated with R software using ggplot2 plugin^56^ (v3.3.5, https://ggplot2.tidyverse.org/).

### In silico assessment of H3K27me3, H2K119Ub and 5mC enrichment at major satellites

To avoid any biases from technical procedures such as mapping of sequence tags, satellites as analysed from ChIP-seq were assayed by counting satellite-containing sequence tags directly from FASTQ sequencing files, and plotting it as percentage from the total number of reads in the FASTQ file^11,57^. The following satellite-specific sequences were used to select for satellite tags: GACGACTTGAAAAATGACGAAATC, CATATTCCAGGTCCTTCAGTGTGC, GAAAAAGGTGGAAAATTTAGA, AGAAAACTGAAAATCATGGAAAAT, GATTTCGTCATTTTTCAAGTCGTC, GCACACTGAAGGACCTGGAATATG, TCTAAATTTTCCACCTTTTTC and ATTTTCCATGATTTTCAGTTTTCT. DNA methylation on satellite repeats was similarly assayed directly from the FASTQ files by determining the percentage bisulfite conversion on C residues within the satellite sequences as listed above. The following datasets were used: GSE73952^20^ for H3K27me3; and GSE76505^23^ for DNA methylation.

### Human or animal rights

All applicable international, national, and/or institutional guidelines for the care and use of animals were followed. This article does not contain any studies with human participants performed by any of the authors.

## Supplementary Information

Supplementary Information 1.

Supplementary Information 2.

Supplementary Information 3.
