# Supplementary material for: H3K27me3 at pericentromeric heterochromatin is a defining feature of the early mouse blastocyst
Source: Sci Rep. 2022 Aug 16;12:13908. doi: 10.1038/s41598-022-17730-x (PMC9381757; doi:10.1038/s41598-022-17730-x)

Supplementary Fig. 1: H3K9me3 at chromocenters in 2-cell to 16-cell stage embryos.

(a) Upper panel row shows the Z-projection of a whole embryo, counterstained with DAPI. Scale bar represents 20µm. Bottom panels show a single section of a representative nucleus highlighted in Figure 1 after staining with DAPI (green) and H3K9me3 (red). Last row is the merge of the two signals. Scale bar represents 5µm for zoomed-in nuclei. **(b)** The graphs show the intensity profiles of DAPI (green), H3K9me3 (red) and H3K27me3 (grey) signals across a single chromocenter highlighted by an arrowhead in **(a)**. **(c)** Violin plots show the distribution of the Pearson's Correlation Coefficients (PCC) between DAPI and H3K9me3 profiles at chromocenters for each stage (149 chromocenters analysed). **(d)** Proportion of tags for H3K27me3 at major satellite sequences during preimplantation stages as assessed from published ChiP-Seq (GSE73952[20]).

Supplementary Fig. 2: H3K9me3 at chromocenters in the peri-implantation embryo, in embryonic compared to extra-embryonic lineages.

(a) Schematic representation of mouse peri-implantation development, lineages specification and their associated markers. **(b, e** and **h)** Embryos at E3.25 **(b)**, E3.75 **(e)**, and E4.25 **(h)** stained for the indicated markers. Upper left panel is a z-projection of the whole embryo showing DAPI counterstaining while other panels are a section encompassing the ICM/EPI region. **(c, f** and **i)** Embryos at E3.5 **(c)**, E4.0 **(f)**, and E5.5 **(i)** stained with H3K9me3. **(d, g** and **j)** Graphs show the representative intensity profiles of DAPI (green) and H3K9me3 (red) signals across a single chromocenter in a nucleus of each lineage at E3.5 **(d)**, E4.0 **(g)** and E5.5 **(j)**.

Supplementary Fig. 3: Correlation dynamics between DAPI and H3K9me3 signals at chromocenters at peri-implantation stages.

(a, c and **e)** Pattern of H3K9me3 in a representative nucleus of EPI **(a)**, PrE **(c)** and TE **(e)** cells at different blastocyst stages (from E3.25 to E4.25). DNA is stained with DAPI. Scale bar represents 5µm. **(b, d** and **f)** Violin plots show the distribution dynamics of the Pearson's Correlation Coefficients (PCC) between DAPI and H3K9me3 profiles at chromocenters in EPI **(b)**, PrE **(d)** and TE **(f)** cells at various stages (196, 192 and 195 chromocenters analysed respectively).

Supplementary Fig. 4: Transcription dynamics of major satellites in 2-cell to 16-cell embryos assessed by RNA-FISH and relative quantification of DNA methylation at major satellites.

(a) Z-projection of whole embryos processed for immuno-RNA-FISH, with DAPI (blue) and Major satellite foci (red) staining. Scale bar represents 10µm. **(b)** Proportion of cells exhibiting 0, 1-2, 3-4 and 5 or more (5+) RNA-FISH foci per cell at each indicated stage (215 nuclei analysed). **(c)**

Proportion of tags for mCpG at major satellite sequences at blastocyst and post-implantation stages as assessed from published DNA methylome (GSE76505[23]).

Supplementary Fig. 5: EZH2 and BEND3 patterns in whole peri-implantation embryos and effects of siEzh2+EPZ experiments on the inactive X.

(a-c) Z-projection of whole embryos at E3.5 **(a)**, E4.0 **(b)**, and E5.5 **(c)** after staining with DAPI, NANOG or OCT4, EZH2 and BEND3. **(d)** Proportion of cells showing 0 to 5 foci after RNA-FISH for Major satellites. **(e, g)** Z-projection of whole female 96h embryos, after electroporation with either scramble or siEzh2 and immuno-stained for DAPI, EZH2 and H3K27me3. EPZ was also added to the culture of siEzh2 treated embryos. The lower panels show a representative nucleus and the line is drawn across the inactive X cloud. **(f, h)** Graphs show the representative intensity profiles of DAPI (green), EZH2 (grey) and H3K27me3 (red) signals across the inactive X cloud of the nucleus shown in **(e)** and **(g)**. **(i)** Quantification of total signal intensity of EZH2 and H3K27me3 within the inactive X chromosome cloud (n=20) of scramble and siEzh2+EPZ treated embryos. *** Pvalue<10^{-9}, Mann-Whitney test.

Supplementary Fig. 6: BEND3 profile at chromocenters in stem cells and transcription dynamics of major satellites in each cell line assessed by RNA-FISH.

(a, b, c) BEND3 staining in 72h outgrowth **(a)**, ESCs **(b)** and cEpiSCs **(c)**. DAPI and NANOG staining are presented in the lower panels. The pluripotent cell population is delineated by a white dashed line in **a**. The proportion (%) of the cell population with BEND3 enriched chromocenters is indicated for each cell population. **(d)** Profile of major satellite transcription (red) assessed by RNA-FISH in 2i/LIF ESCs, cEpiSCs and FAXY TSCs. The proportion of cells exhibiting RNA-FISH foci is specified for each cell line in the zoomed-in nucleus panel (1297, 674 and 1286 nuclei analysed respectively). **(e, f)** FAXY-cultured TSCs colony stained for H3K27me3 **(e)** or BEND3 **(f)**, DAPI and merge signals. **(g)** A representative intensity profile graph of DAPI (green), BEND3 (grey) and H3K27me3 (red) signals across a single chromocenter in a nucleus of TSCs. **(h, f)** Relative expression of genes associated with primed (left) or naïve (right) pluripotency during the conversion of ESCs to EpiSCs presented in **Fig. 7g**.

Supplementary Fig. 7: Representative dynamics of the epigenetic profile of chromocenters along peri-implantation development.

This scheme summarizes the main findings of the study: the epigenetic organization of a representative chromocenter at different stages and in embryonic and extra-embryonic lineages, as well as in different stem cells for better comparison: the histone modifications (H3K27me3 and H3K9me3) are present either in the core region (circle) or at the periphery (ring). Associated proteins

(EZH2 and BEND3) present at PCH are represented by coloured rectangles when present. The checkerboard pattern indicates the presence of the two PTM or the two proteins. DNA methylation at major satellites is represented by stars (white: unmethylated; black filling: methylated). ND: unknown data

Supplementary Figure 1

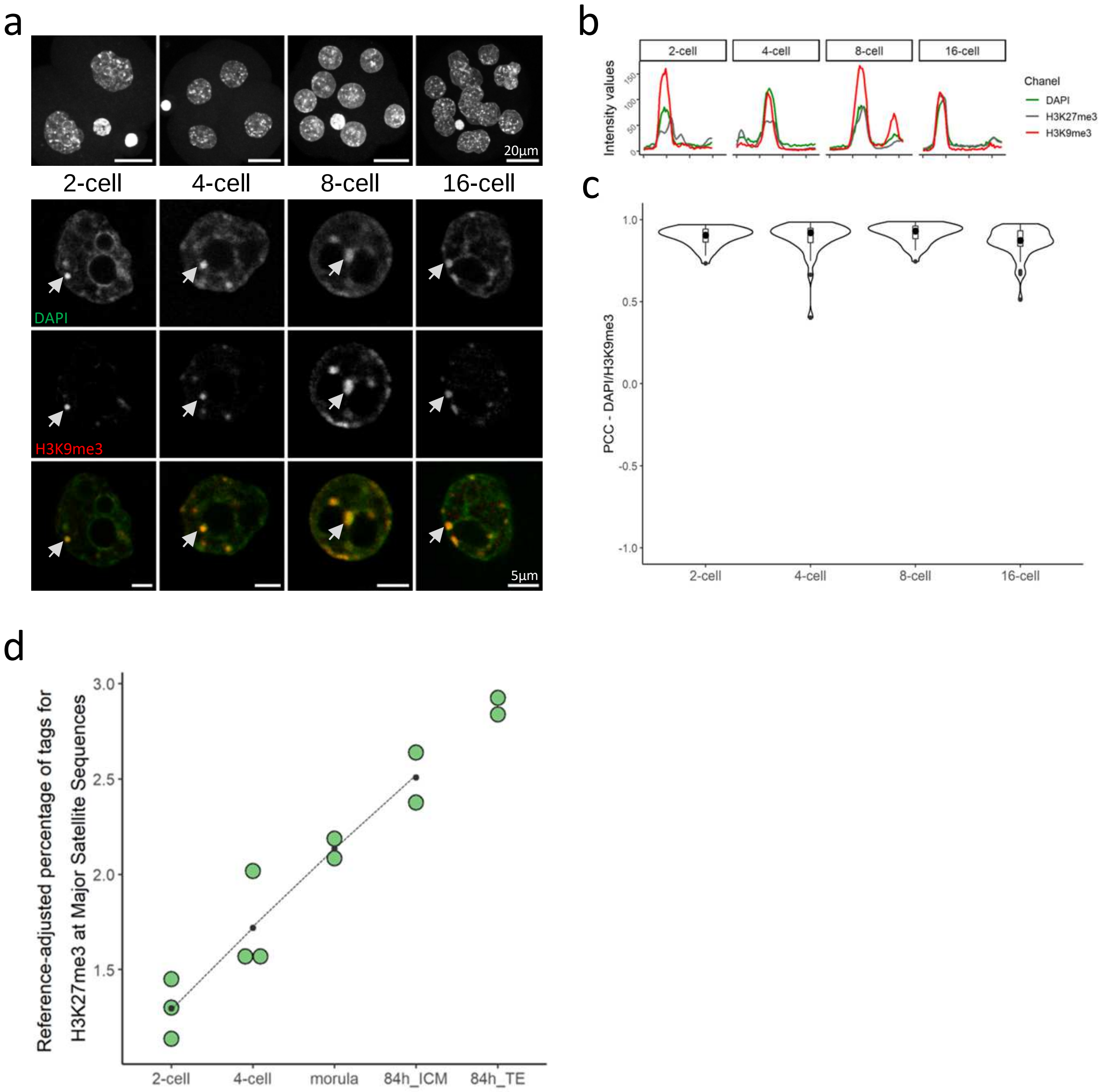

Supplementary Figure 2

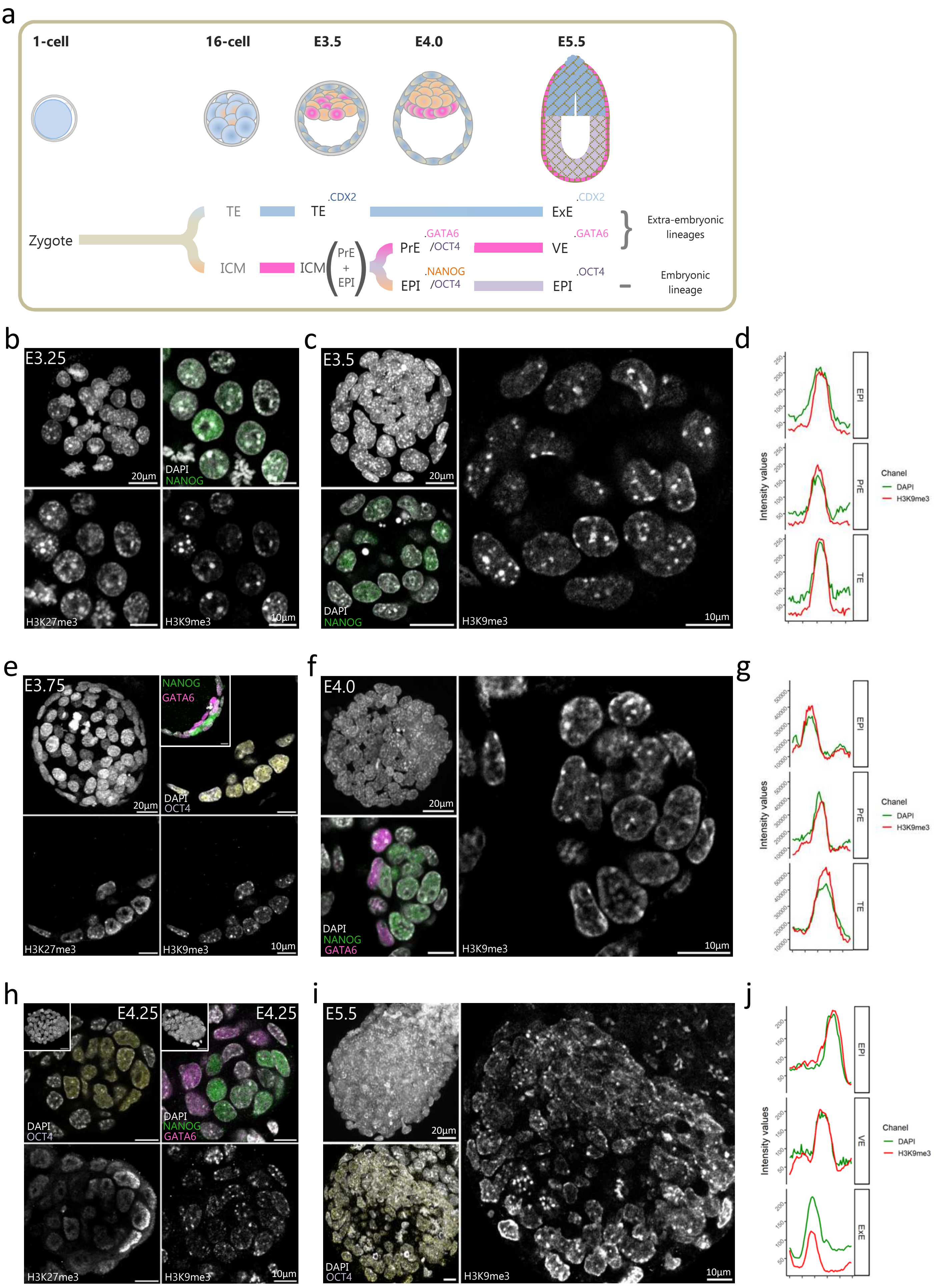

Supplementary Figure 3

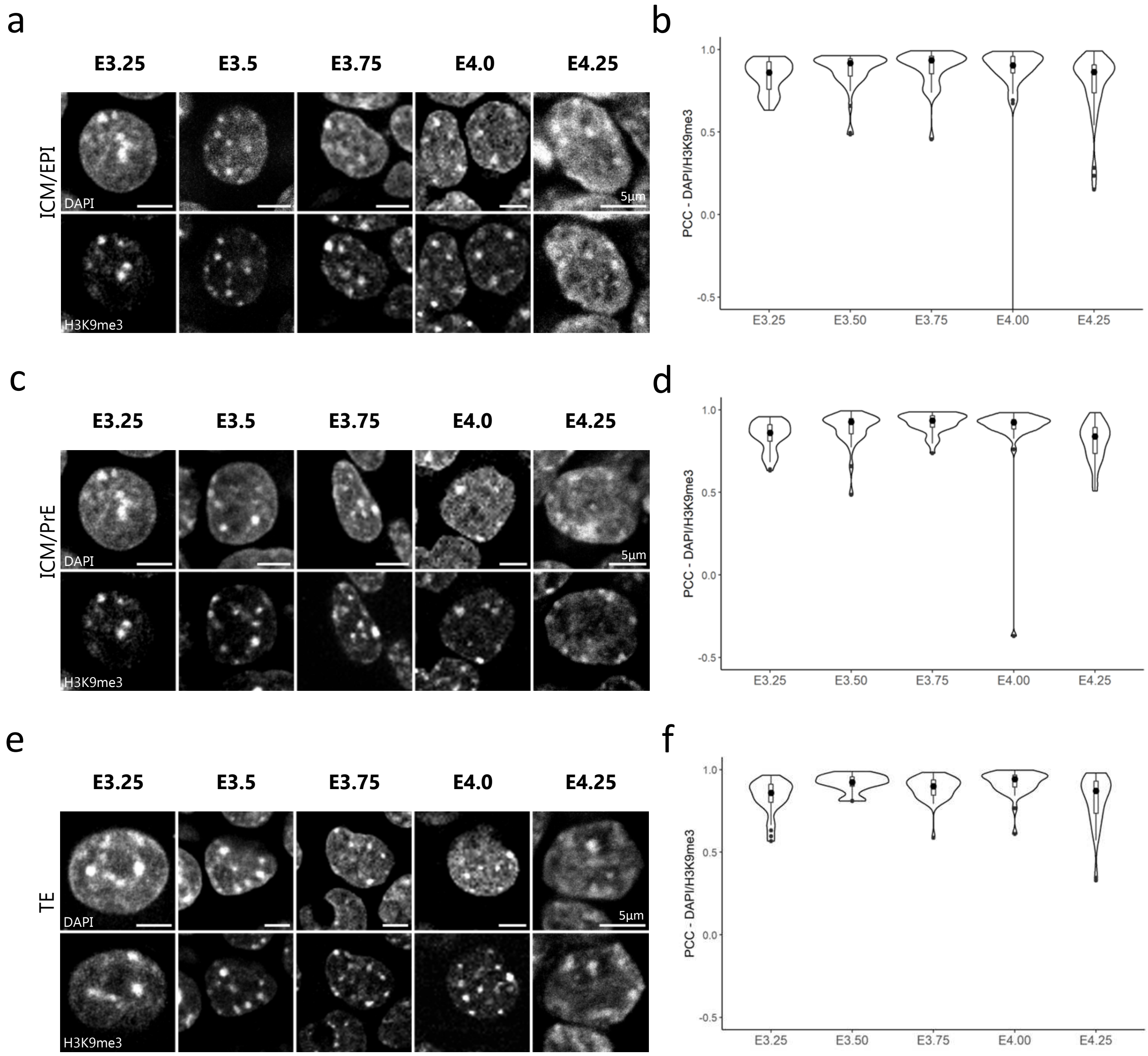

Supplementary Figure 4

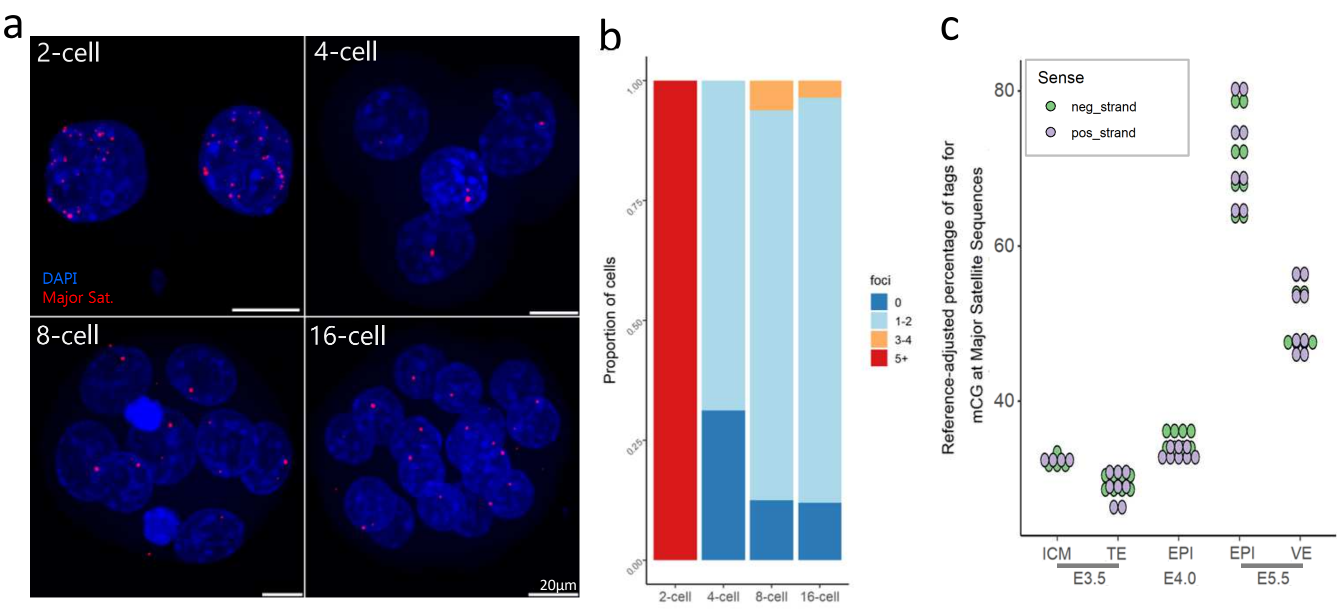

Supplementary Figure 5

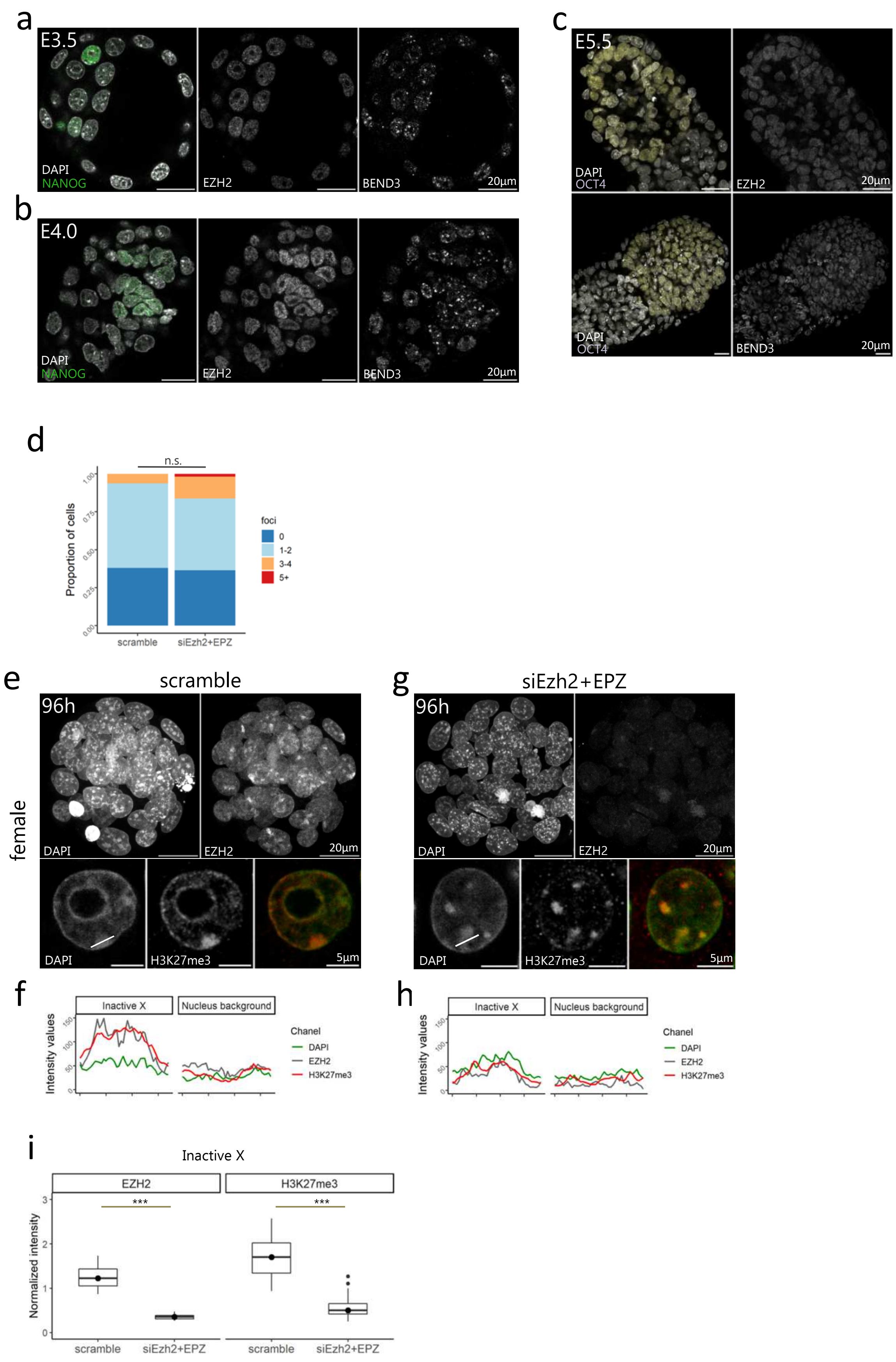

Supplementary Figure 6

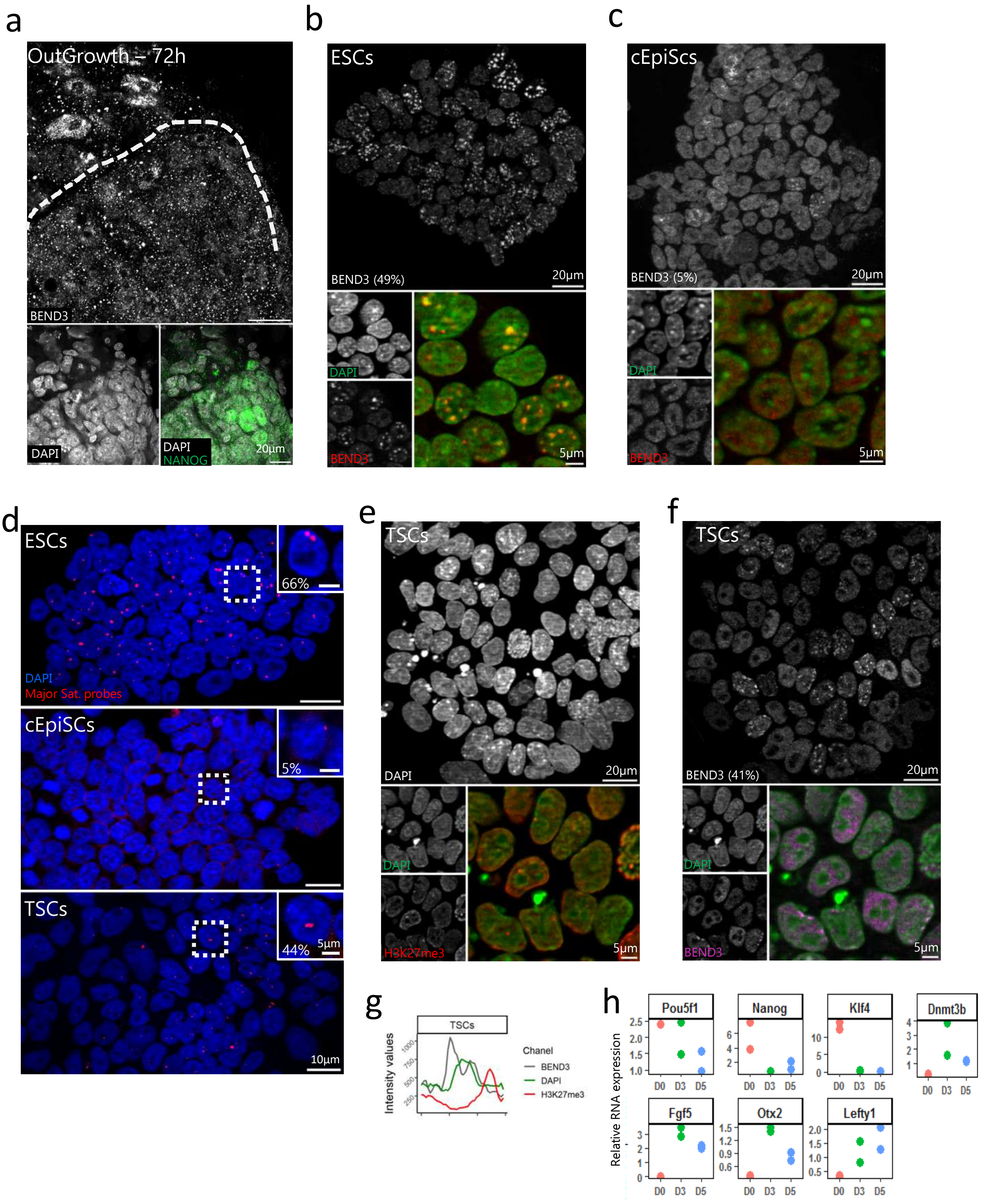

Supplementary Figure 7

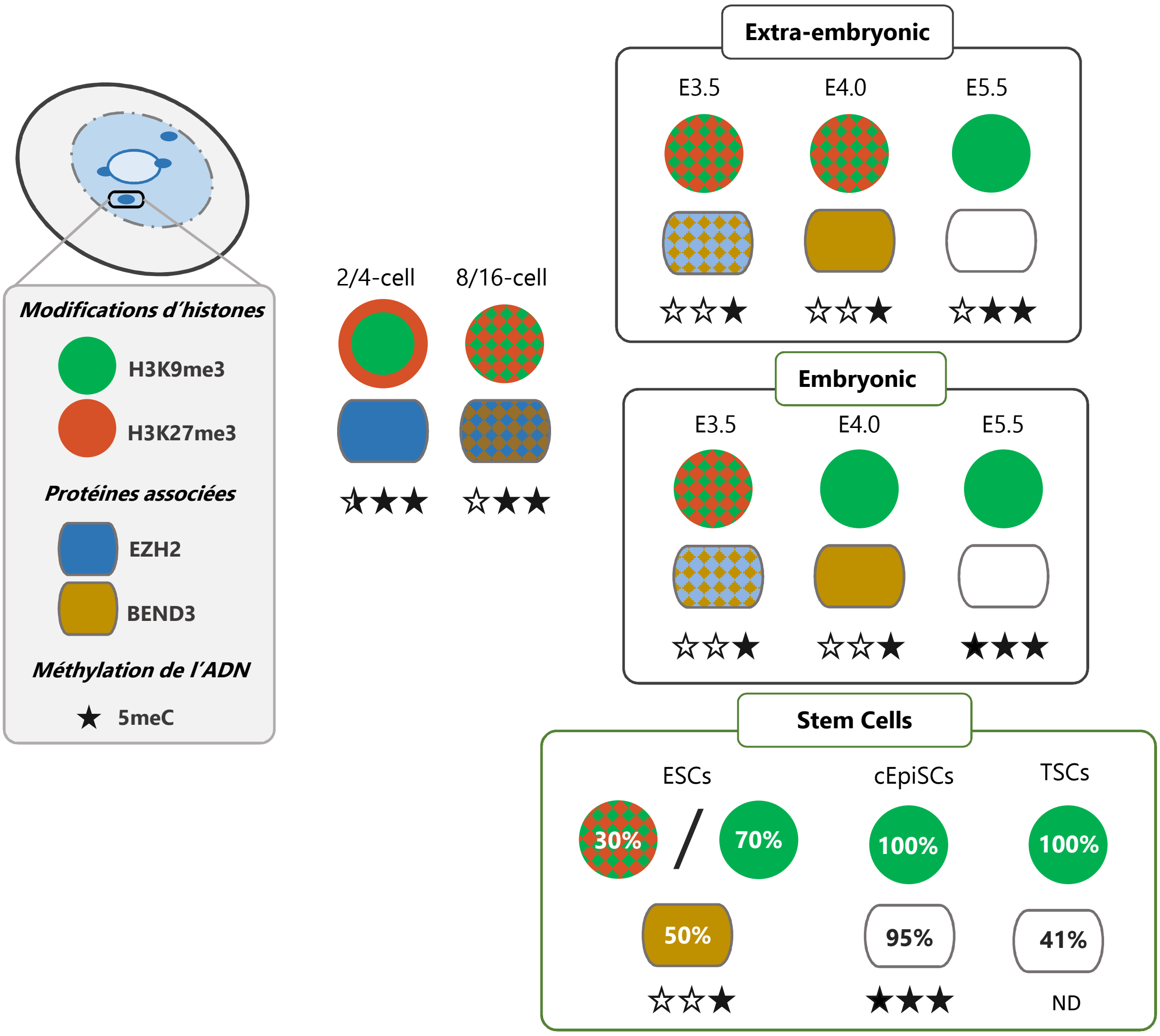

Supplement: Supplementary file 1 — Supplementary Information 1. [file 41598_2022_17730_MOESM1_ESM.pdf]
